# A Gated Multi-Objective Learning-to-Optimize Framework for Fault-Tolerant Sensor Placement in Bridge Structural Health Monitoring

**DOI:** 10.3390/s26216915

**Published:** 2026-10-31

**Authors:** Oumayma Najem, Mohammed Benbrahim, Mohammed Nabil Kabbaj, Jaouad Boumhidi

**Affiliations:** 1 Systems Engineering, Modeling and Analysis Laboratory, Faculty of Sciences, Sidi Mohamed Ben Abdellah University, Fez 30500, Morocco; mohammed.benbrahim@usmba.ac.ma (M.B.); n.kabbaj@usmba.ac.ma (M.N.K.); 2 L3IA Laboratory, Faculty of Sciences, Sidi Mohamed Ben Abdellah University, Fez 30500, Morocco; jaouad.boumhidi@usmba.ac.ma

**Keywords:** structural health monitoring, optimal sensor placement, learning-to-optimize, multi-objective optimization, fault tolerance

## Abstract

Structural Health Monitoring (SHM) relies on sensor placement to collect structurally relevant data, which are then interpreted according to the monitoring objective. Optimal Sensor Placement is therefore an essential step for collecting useful information for the monitoring task. However, conventional SHM design often separates the OSP and interpretation steps. Additionally, OSP approaches generally require a separate optimization process for each structural case. This study proposes L2O-OSP, a learning-to-optimize framework that learns sensor placement decisions across a population of bridge configurations. The architecture contains three components: a gating network that determines the sensor budget and the relative weights of the placement objectives; a selector network that ranks candidate response locations and retains the top-k candidates; and a prediction network that estimates severity from the selected structural responses. The placement objective combines prediction error, spatial coverage, sensor count, and a robustness-related term. The framework is evaluated using 2000 parametrically generated bridge configurations and three numerical loading cases per configuration. The model trained without sensor failures selected an average of 74.40 sensors from 180 candidate locations, a reduction of 58.67%, and achieved a severity MAE of 0.02389 with all selected sensors available. At 50% test sensor failure, its MAE increased to 0.03557 without retraining. We compare global severity-oriented ranking with L2O-OSP at matched sensor count; both methods achieved similar prediction errors, but L2O-OSP reduced the mean largest uncovered interval from 9.56% to 3.09% of the bridge span. Additional evaluations examined Gaussian measurement noise, candidate-count sensitivity, and generalization to bridge lengths outside the training range. These findings support the proposed multi-objective approach within the evaluated numerical setting.

## 1. Introduction

The constant exposure of structures to combinations of different load types, environmental stresses, and material fatigue eventually leads to structural changes that are not always visible on the surface. As a result, periodic visual inspections are rarely enough to detect all existing damage. Structural Health Monitoring (SHM) refers to the assessment of civil infrastructure conditions through continuous measurements of structural response [1]. From a monitoring perspective, SHM can be viewed as a two-step system consisting of, first, the acquisition of the structural responses through a sensor network and the subsequent analysis of the acquired data to assess the structural condition. Sensor placement is therefore a critical part of the data-acquisition stage, since the number and location of sensors determine the quality of information available for the following structural assessment step. Although dense networks can provide exhaustive measurements, practical constraints such as installation, acquisition, and cost limit the number of sensors that can be deployed. Optimal Sensor Placement (OSP) consequently aims to identify a reduced sensor configuration that collects information that is useful to the structural assessment task [2,3,4,5,6,7]. Chioccarelli et al. [8] showed that four optimally placed accelerometers achieve the same identification capability as 30 sensors on an existing bridge.

OSP is fundamentally a combinatorial optimization problem in which a subset of sensor locations is selected from a larger set of candidate locations [9]. Different families of optimization methods have been developed to address this problem, ranging from exact and approximate methods to heuristic and, more recently, learning-based approaches [10]. Exact optimization methods systematically explore the solution space and provide an optimal solution. However, their computational requirements increase with the number of candidate locations. Approximation methods reduce the computational expenses by simplifying the original optimization problem, consequently resulting in deviations from the global optimum [11]. These limitations are addressed by iterative analytical and heuristic approaches that provide solutions without exhaustively evaluating the complete search space [12].

Among analytical OSP approaches, modal information-based criteria such as Effective Independence (EI) identifies sensor locations based on their contribution to the linear independence of the target mode shapes [13,14]. Sun et al. [15] showed that EI can be used for sensor placement to meet monitoring requirements while reducing cost. Chai et al. [16] improved the robustness of mode selection of EI-based optimal placement by integrating a sensitivity measure related to structural damage. Meo and Zumpano [17] showed that the Effective Independence Driving Point Residue (EFI-DPR) preserved the information required to characterize the bridge vibration modes while maintaining adequate signal strength under measurement noise. Li et al. [18] investigated the relationship between EI and Modal Kinetic Energy (MKE) using the I-40 Bridge in New Mexico. They showed that EI could be interpreted as an iterative extension of MKE in which the reduced mode shapes are repeatedly re-orthonormalized during sensor elimination. However, their solutions are driven by predefined analytical objectives, which can limit their ability to consider several deployment requirements simultaneously.

To adjust to multiple objectives and constraints, heuristic and metaheuristic optimization methods have been used for OSP. Population-based optimizations such as Genetic Algorithms (GA) formulate sensor placement as a combinatorial optimization problem that can accommodate different placement objectives and constraints. Evolutionary and population-based algorithm optimization methods are widely used to solve the OSP combinatorial problem and have been validated on realistic structures [19,20,21]. These approaches can optimize multiple objectives, including cost, coverage, and fault-tolerance. Civera et al. [22] used a multi-objective genetic algorithm to improve the robustness of sensor configurations under potential structural deterioration. Saleem and Jo [23] proposed a multi-objective sensor placement optimization framework for bridge structures that use heterogeneous sensor types in spatially varying loading conditions. Cha et al. [24] used a multi-objective genetic algorithm framework to optimize sensor placement in seismically excited structures to simultaneously minimize structural dynamic response and optimize device location. He et al. [25] combined modal strain energy with an adaptive genetic algorithm to optimize both the number and locations of sensors on a bridge structure, using modal-information to guide the search. Zhang et al. [26] proposed a modified genetic algorithm, Meta-GA, for multi-objective sensor placement on the Lugou Bridge. The method improved the computational efficiency and damage sensitivity of the selected configuration compared with conventional GA based approaches.

However, heuristic and metaheuristic methods are iterative search processes performed independently for each optimization instance. When structural characteristics, candidate locations, monitoring conditions, or design requirements change, the optimization process needs to be repeated. Although these approaches are very effective for individual structures, they become computationally expensive when placement decisions must be regenerated across different structures with varying conditions. Learning-to-optimize (L2O) is a machine-learning model that learns how to solve optimization problems from a population of optimization instances and then generates solutions for new, unseen instances without solving each optimization problem independently [27,28]. In OSP, this creates the opportunity to learn how structural characteristics and load conditions relate to the sensor layouts.

Learning-based approaches have been introduced for sensor placement in SHM. Meng et al. [29] used deep reinforcement learning (DRL) to optimize vibration sensor placement based on the modal criterion (MAC), modeling the placement process as a Markov decision process and achieving better placement performance than genetic and particle swarm optimization algorithms. Sajdi and Liang [30] combined deep generative models with Bayesian optimization for sensor placement in data-driven SHM models. Shi et al. [31] used an attention-based neural network to identify sensor locations relevant to a damage detection task. Zhong et al. [32] proposed a heuristic-enhanced deep reinforcement learning framework for multi-type sensor placement, combining heuristic exploration with a Bayesian information-based reward function to improve the placement optimization strategy. These studies show different learning-based approaches to sensor placement, such as reinforced learning, Bayesian optimization, and attention-based selection. Our proposed method L2O-OSP builds on this research direction by jointly learning bridge-specific sensor counts and locations.

However, learning a sensor placement strategy involves more than determining a fixed set of sensor locations. The effectiveness of a sensor configuration is evaluated by the relevance of the acquired information to the monitoring objective, its cost efficiency, its coverage of the structure, and tolerance to sensor failures and malfunction. This encourages a direct connection between sensor placement and damage assessment stages by integrating monitoring performance into the placement decision. The present work investigates this idea in a model-based numerical setting [33,34,35,36,37,38]. Adaptive conditioning has been explored in other prediction tasks. PCIM-DTA uses pair-specific features to modulate interaction [39], while SIGMA-DTA uses similarity to the training distribution to weight interaction paths [40]. In our proposed framework L2O-OSP, bridge characteristics guide sensor counts and objective weights.

Our study proposes L2O-OSP, a learning-to-optimize framework for damage-oriented sensor placement in bridge SHM. The framework learns sensor placement decisions across parametrically varying bridge configurations through three interconnected components. A gating network determines the sensor budget and balances the placement objectives, a selector network uses FE-generated responses at all candidate locations to score and select locations for each bridge, and a prediction network estimates the minimum retained stiffness factor from the selected responses. The selected configurations are assessed through a multi-objective formulation that considers prediction performance, sensor number, spatial coverage, and fault tolerance. The framework generates bridge dependent sensor configurations, avoiding the need for new iterative optimizations for every bridge configuration. Separate models are trained for prescribed sensor failure rates, and cross-failure testing evaluates their performance without retraining.

The main contributions of this study are: (1) An L2O-based framework is introduced to generate sensor configurations across varying structural parameters. The framework uses structural and loading characteristics to determine a bridge-dependent sensor count and objective weighting, avoiding the need to independently perform an iterative optimization procedure for each bridge configuration. (2) The proposed architecture combines a gating network, which determines the sensor budget *k* and relative weights, with a selector network that scores the candidate sensor locations and retains the *k* highest ranked locations through a hard top-k operation. (3) The selected sensor configurations are evaluated with respect to the damage severity prediction task through a multi-objective formulation that additionally considers spatial coverage, sensor deployment cost, and fault tolerance.

This study aims to select bridge-specific sensor layouts that balance severity-prediction error, sensor count, spatial coverage, and prediction under sensor failures. The main challenge is to reduce the number of sensors while maintaining useful response information and limiting large, uncovered regions. The framework is evaluated using simulated bridge responses, with additional tests under changing sensor availability, measurement noise, and bridge lengths outside the training range. Experimental deployment and sensor communication hardware are outside the present scope.

The remainder of this paper is organized as follows: Section 2 presents the numerical bridge dataset and the proposed L2O-OSP methodology, including the gating, selector, and prediction networks and their training objectives, as well as the experimental setup and evaluation procedure. Section 3 presents scenario-specific results, cross-failure evaluation, baseline comparisons, length OOD evaluation, candidate-count sensitivity, and measurement noise testing. Section 4 discusses the findings and limitations of the proposed framework, and Section 5 concludes the study.

## 2. Materials and Methods

This section presents the L2O-OSP framework for bridge-dependent sensor placement. L2O-OSP is a model-based sensor network design framework that uses bridge characteristics and Finite Element (FE) structural responses to determine a sensor configuration before deployment. Candidate locations are sampled uniformly along the finite element mesh. Each candidate represents an element-level response location, with horizontal displacement, vertical displacement, and rotation extracted at its two end nodes. The six response components are collected for the static, traffic, and wind loading cases, giving 18 features per candidate. The selected configurations determine which candidate responses are retained for severity prediction.

**Figure 1:**
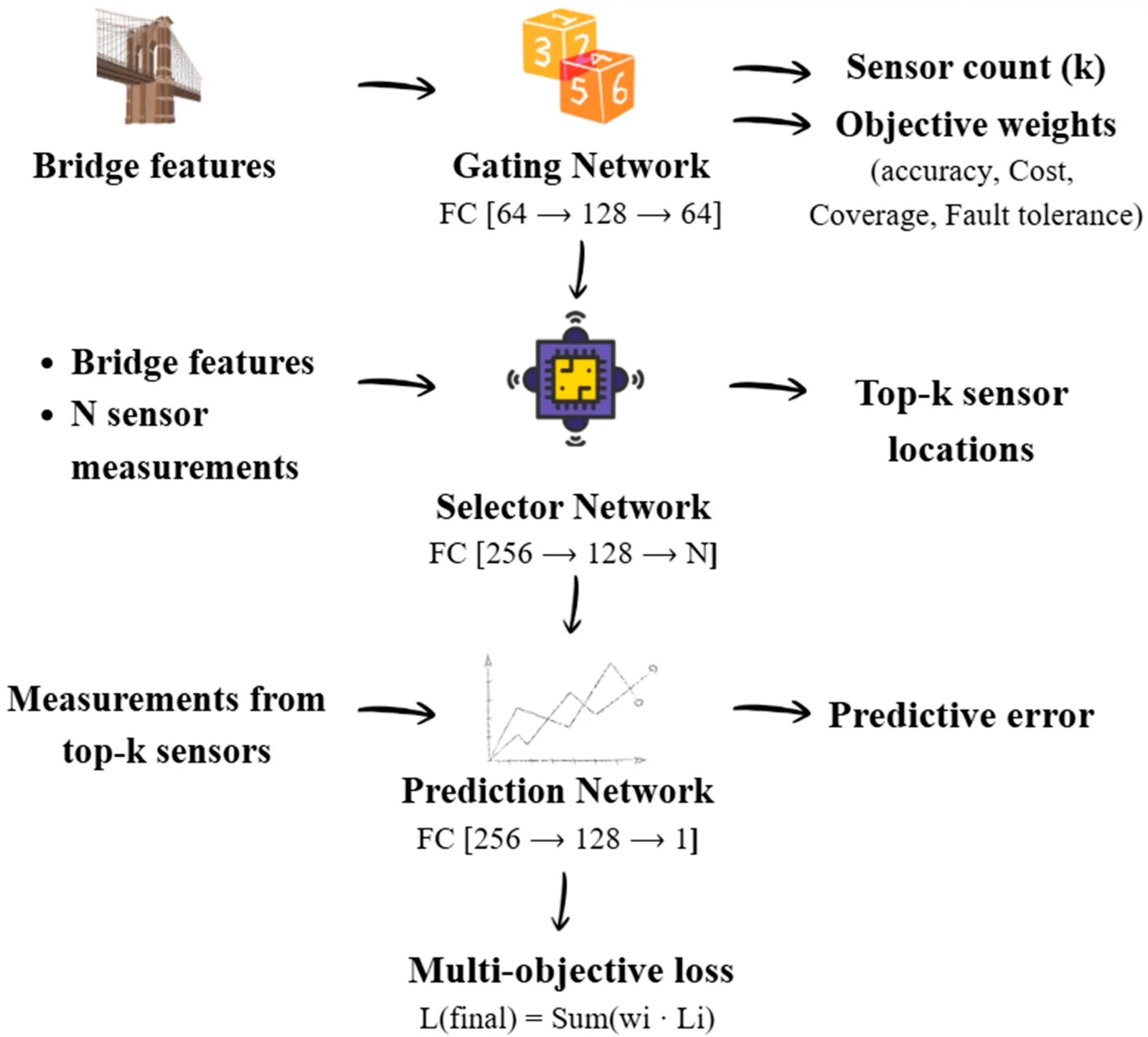
L2O-OSP framework overview.

The framework consists of three interconnected networks: A gating network that controls sensor count and the weights of each optimization objective. A selector network that identifies the most informative locations on the structure. A prediction network that uses the measurements chosen by the selector network to estimate damage severity. Figure 1 provides an overview of the complete methodology. The framework was implemented in Python using PyTorch (version 2.11.0).

### 2.1. Problem Formulation

Consider a bridge structure discretized into N candidate sensor locations, where each location *i* has FE-generated response features associated with an element and its two end nodes. The goal of optimal sensor placement is to identify a subset *S* ⊂ *{1*, *2… N}* of *k* sensor locations that balances severity prediction error, spatial coverage sensor count, and prediction under sensor failures, subject to allowable sensor count bounds:

*min L_Total_*,   subject to *k_min_* ≤ |*S*| ≤ *k_max_* (1)

where *L_Total_* is the training objective defined in Section 2.7, and *k* = |*S*| is the selected sensor bound. Cost is assumed proportional to sensor count throughout this paper.

### 2.2. Data Generation

Training an L2O framework requires a sufficiently diverse dataset to ensure that the learned placement policy generalizes to unseen structures. To achieve this, we generated a dataset of 2000 unique bridge configurations using OpenSees through its Python interface, OpenSees (version 3.8.0.0) [41], with selected parameter ranges informed by Japanese Highway Bridge design specifications [42]. Each bridge was modeled as a two-dimensional beam, with the left-end node fully restrained and the vertical displacement restrained at the right-end node. The geometric, structural, and loading parameters were varied across configurations as summarized in Table 1.

**Figure 2:**
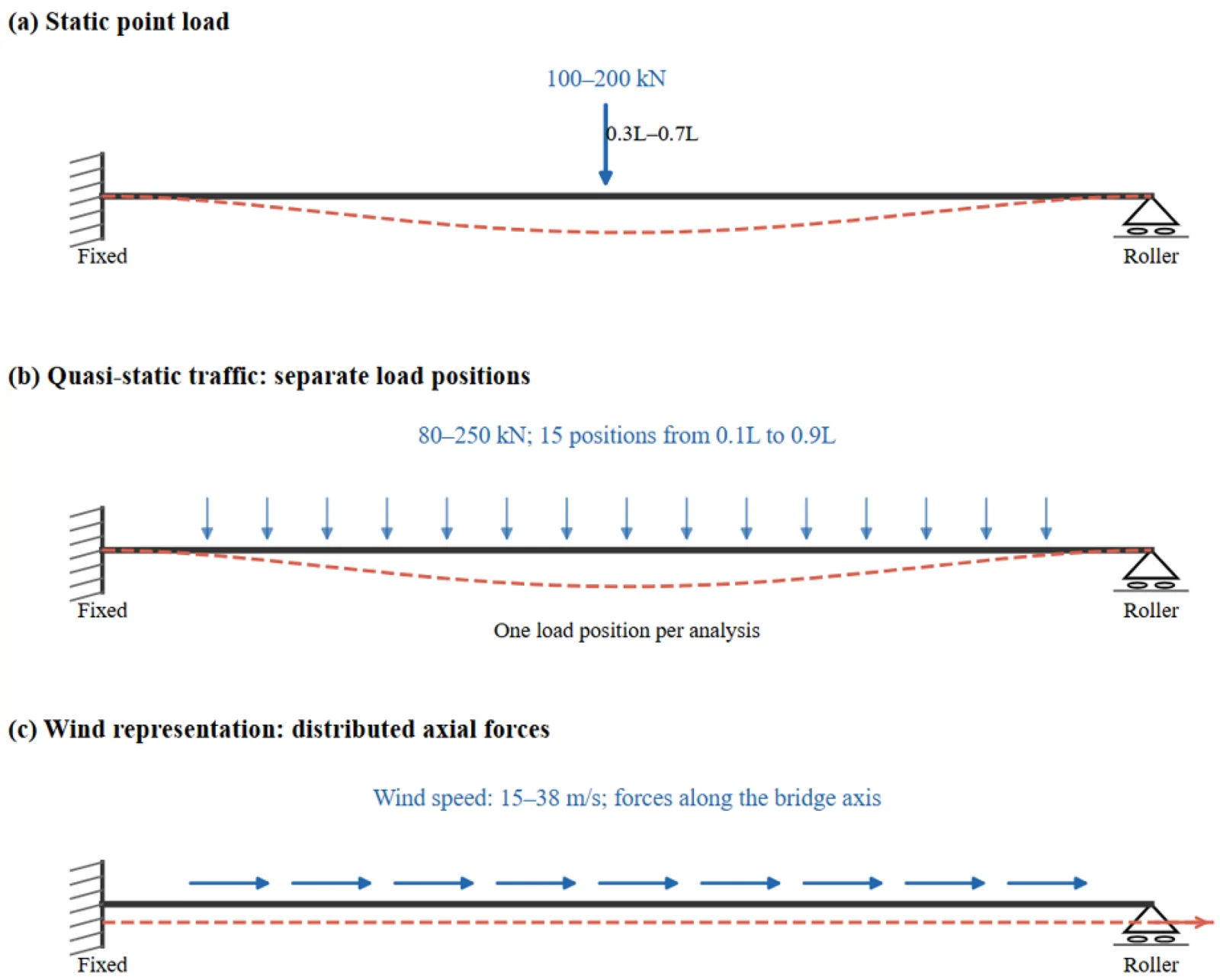
Loading cases applied to the bridge model: (**a**) static loading, (**b**) quasi-static traffic at 15 positions, and (**c**) distributed axial forces representing wind.

Each finite-element response contains six components corresponding to the horizontal displacement, vertical displacement, and rotation at the two end nodes of the associated element. The 800–1500 finite elements define the numerical resolution used to generate structural responses. For the sensor placement framework, *N* = 180 candidate locations are sampled uniformly across the full finite element mesh of each bridge. Each candidate location is represented by the six response components under each of the three prescribed loading cases: static, traffic, and wind, as shown in Figure 2.

**Figure 3:**
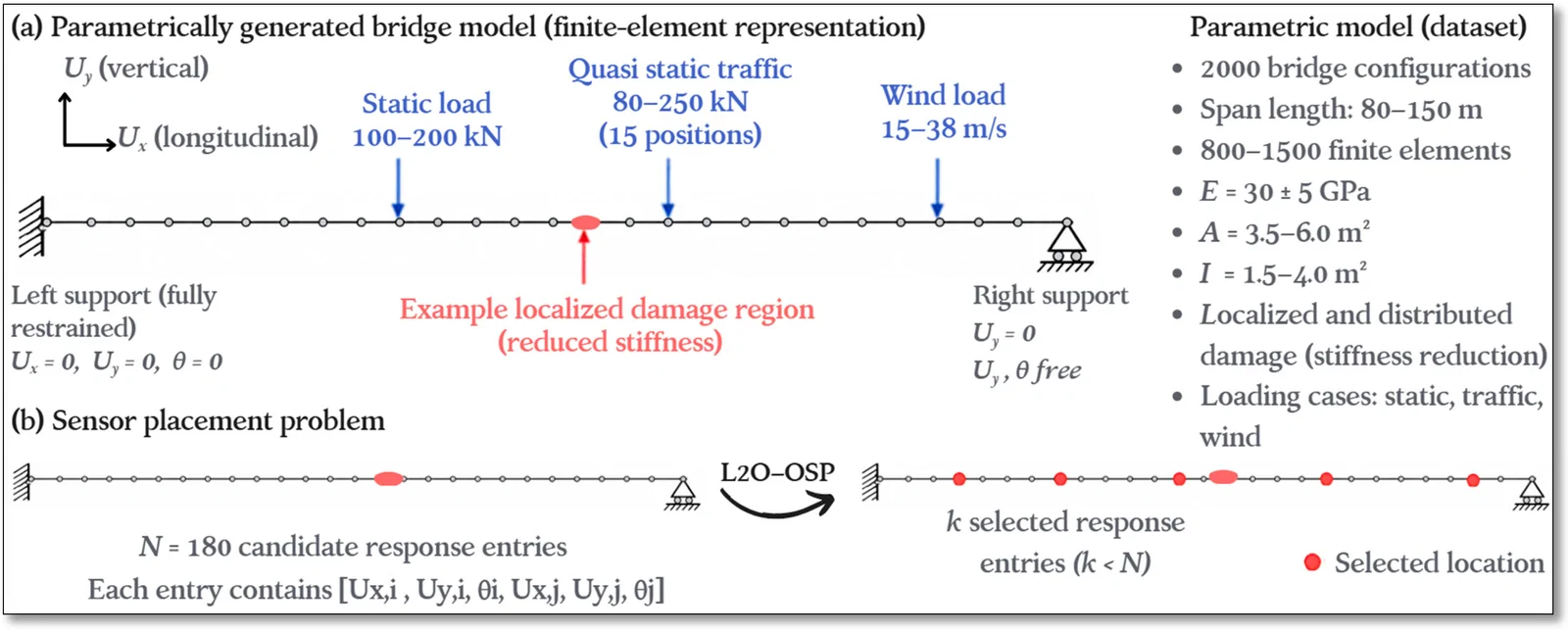
Numerical bridge model and sensor placement formulation.

Three loading conditions are considered: static, quasi-static traffic, and wind loading. The static point load is applied at a randomly selected position between 0.3 L and 0.7 L. Traffic loading is represented by vehicle loads applied sequentially at 15 positions along the bridge span. Wind loading is represented by axial nodal forces along bridge axis. Structural damage is simulated through reductions in element stiffness. Localized damage is introduced within a region spanning 5–15% of the elements, whereas distributed damage is applied across the structure. The localized and distributed stiffness factors range from 0.6 to 1.0 and 0.7 to 1.0, respectively. These ranges represent severe localized stiffness loss and moderate stiffness degradation distributed across the structure. A ±2% perturbation is additionally applied in the localized cases to represent material variability. The prediction target is the minimum retained stiffness factor across all elements, $d=\min_{e}(\frac{E_{e}}{E_{0}})$, where E*_e_* and E_0_ are the damaged and original elastic moduli. This definition applies to both localized and distributed damage and represents the largest element-level stiffness loss. Figure 3 illustrates the numerical bridge model, the candidate response locations, and the sensor placement problem formulation.

### 2.3. Network Architecture

**Figure 4:**
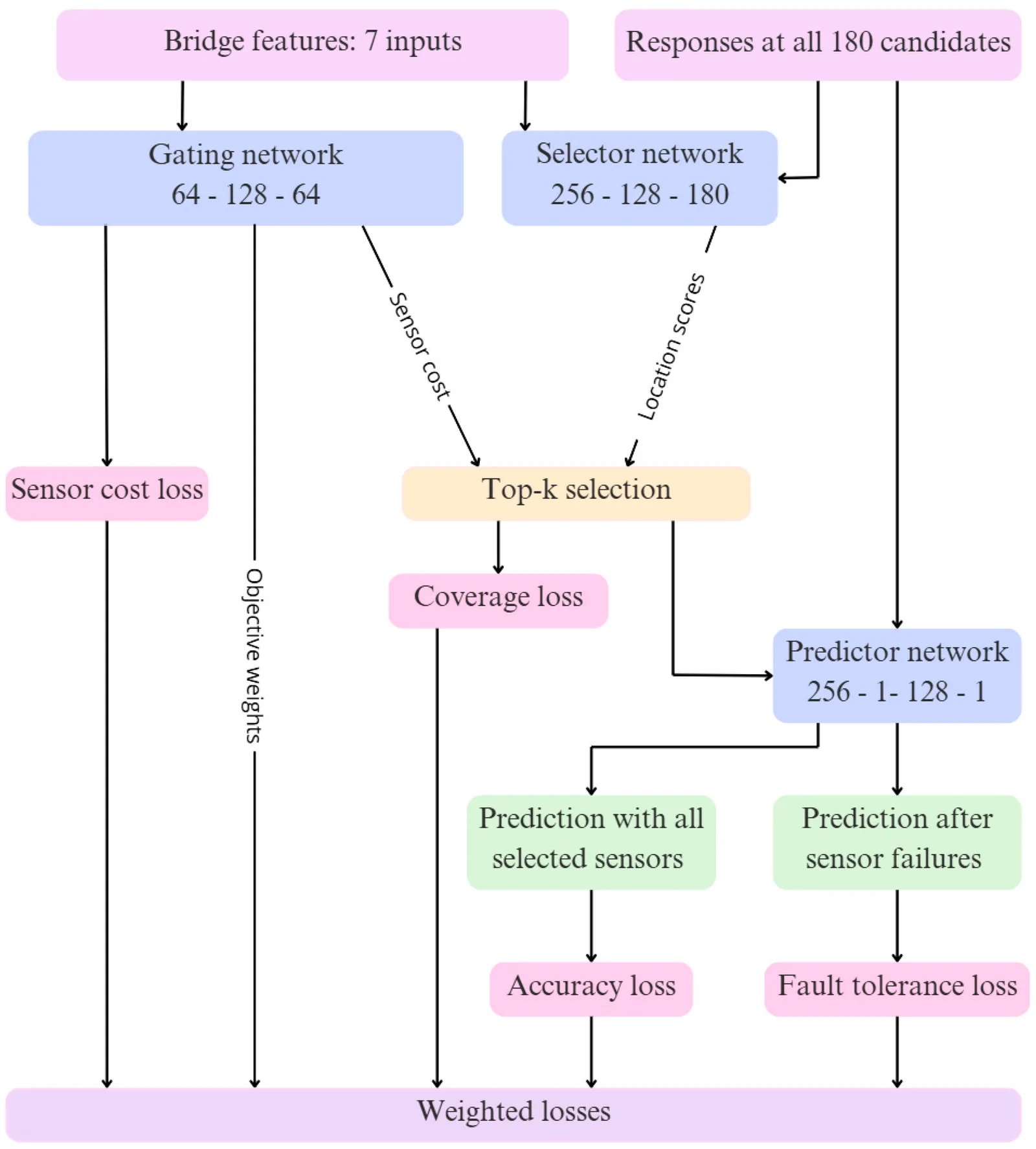
L2O-OSP detailed architecture.

Effective sensor placement requires decisions at two distinct levels: determining the number of sensors and identifying their locations. L2O-OSP addresses these through three interconnected networks, illustrated in Figure 4. The gating decides the sensor number and objective weights, both of which adapt to structural characteristics and training scenario. The selector network assigns a score to each candidate location and retains the *k* highest ranked locations through a hard top-*k* operation. Finally, the prediction network evaluates the damage severity from measurements of the nodes selected by the selector network. The three networks are described in detail in the following sections.

### 2.4. Gating Network

**Figure 5:**
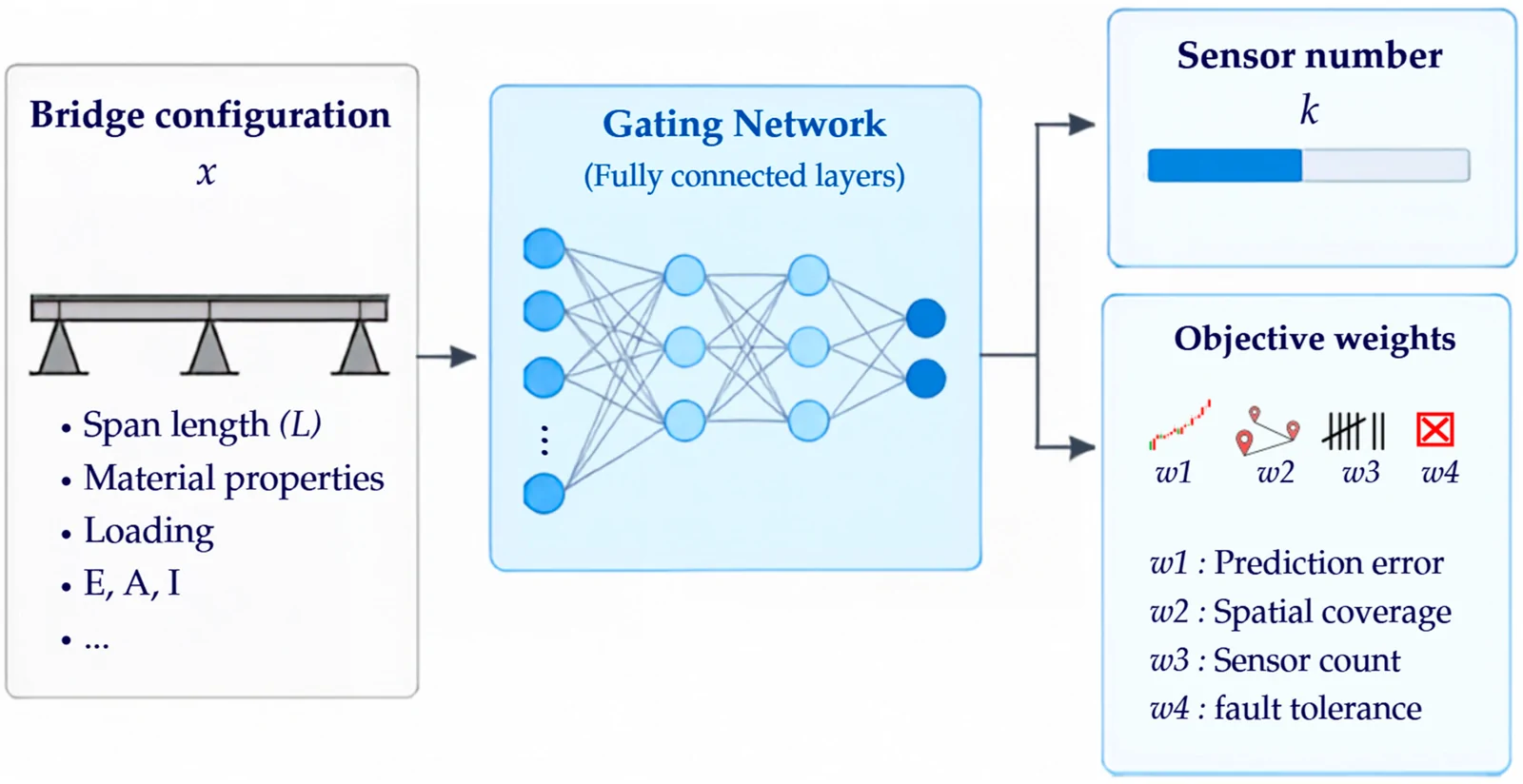
Gating network: sets sensor number and objective weights.

The gating network inputs are the geometric, loading, and material properties of the bridges in the dataset. The network outputs two decisions: the number of sensors *k* and the relative weights *w_i_* assigned to the optimization objectives based on each bridge’s structural and loading characteristics. Sensor failure rate is a scenario-specific training setting rather than a gating input. Measurement noise is evaluated during testing without retraining. The architecture consists of three fully connected layers with 64, 128, and 64 neurons, ReLU activations, and a dropout rate of 0.2. The objective weight head uses softmax followed by rescaling to produce four objective weights that sum to one, each with a minimum value of 0.05. The sensor-count head uses a sigmoid function to produce a continuous sensor number between 5 and N. This value is rounded to determine the number of locations retained by hard top-k selection. During training, a differentiable surrogate allows the prediction loss to update the sensor-count head and selector. The input, architecture, and outputs of the gating network are illustrated in Figure 5.

### 2.5. Selector Network

**Figure 6:**
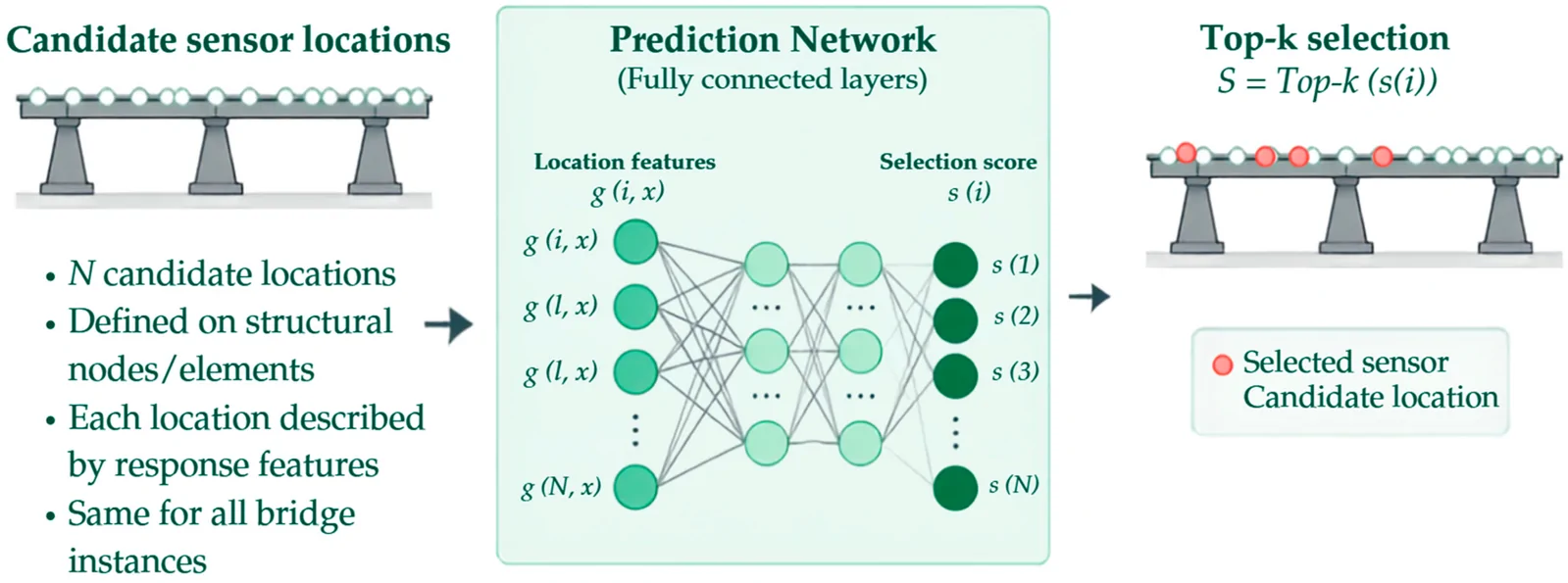
Selector Network: ranks and selects sensor locations.

The selector network receives the bridge features and the FE-generated structural responses at all N candidate locations. These inputs are concatenated and passed through three fully connected layers with 256, 128, and N neurons. ReLU activations and dropout at a rate of 0.2 are applied after the first two layers. The selector network assigns an importance score to each candidate location. It selects the k highest scoring locations using the sensor number from the gating network. During training, the forward pass uses hard top-k selection, while a sensor-count-constrained sigmoid surrogate with temperature 0.5 supplies gradients to the location scores and continuous sensor count. During evaluation, selection uses only the hard top-k. The subsequent prediction network uses only the available responses at the selected locations. Figure 6 provides the selector network overview. Here, *g*(*i*, *x*) is the response features at candidate location *i* for bridge configuration *x*.

### 2.6. Prediction Network

**Figure 7:**
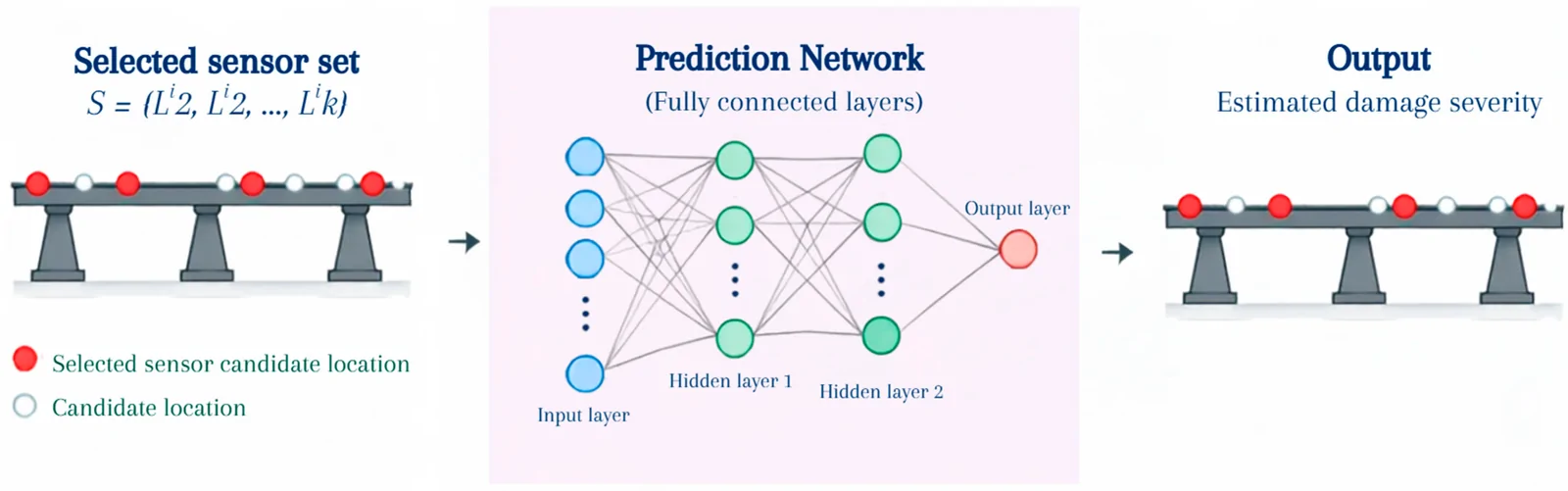
Prediction network: Estimates damage severity from the selected sensors.

The prediction network estimates the minimum retained stiffness factor from the available responses at the selected locations. Each candidate retains a fixed position in the input, ordered along the bridge span. Unavailable responses are set to zero. The predictor receives the responses and a binary availability indicator for each candidate location. The network consists of fully connected layers with 256 and 128 neurons, followed by an output layer with one neuron representing the predicted damage severity. The prediction network architecture is explained in Figure 7.

### 2.7. Multi-Objective Loss Function

A sensor configuration must satisfy multiple criteria such as preserving information, providing spatial coverage, limiting the number of sensors, and maintaining performance under sensor failures. Accordingly, four criteria are considered in the proposed framework: prediction accuracy, spatial coverage, sensor count cost, and fault tolerance.

*L_Total_* = *w_accuracy_* ⋅ *L_accuracy_* + *w_coverage_* ⋅ *L_coverage_* + *w_cost_* ⋅ *L_cost_* + *w_fault_* ⋅ *L_fault_* (2)

- The prediction loss evaluates the accuracy of damage severity estimation and is defined as the squared difference between the true and predicted damage severity values:

(3) $$L_{\mathrm{accuracy}}={\left(d-\hat{d}\right)}^{2}$$

- We evaluate the spatial coverage of the selected sensor layouts using the sum of squared gaps between consecutive selected locations, including the intervals at both ends of the bridge:

(4) $$L_{\mathrm{coverage}}=\sum_{j=0}^{k}{{(x}_{j+1}-x_{j})}^{2}$$

where *x*_1_, …, *x_k_* are the selected locations in ascending spatial order, normalized to [0, 1], with *x*_0_ = 0 and *x_k_*_+1_ = 1. For a fixed sensor count, this criterion favors evenly distributed sensor locations and penalizes large uncovered regions.

- The sensor cost is represented by a sensor count surrogate. This is based on the assumption that installation, cabling, data acquisition, and the maintenance effort scale approximately with the number of deployed sensors, so that sensor count serves as a practical proxy for the overall deployment cost. The corresponding criterion is defined as:

(5) $$L_{\mathrm{cost}}=\frac{k}{N}$$

where *k* is the number of selected sensors and N is the total number of candidate locations.

- The fault-tolerance loss evaluates severity prediction when a prescribed fraction of the selected sensors is unavailable. During training, we randomly remove selected sensors according to the scenario’s failure rate. Their responses and availability indicators are set to zero, and the same prediction network estimates severity from the surviving measurements. The fault-tolerance loss is the squared error between this prediction and the target minimum retained stiffness factor.

(6) $$L_{\mathrm{fault}}={\left({\hat{d}}_{\mathrm{fault}}-d\right)}^{2}$$

where ${\hat{d}}_{\mathrm{fault}}$ is the severity prediction from the surviving selected sensors after simulated failures, and *d* is the target minimum retained stiffness factor.

The accuracy loss evaluates prediction with all selected sensors available, whereas the fault-tolerance loss evaluates prediction after simulated sensor failures.

### 2.8. Training Procedure

The 2000 bridge configurations are divided into training, validation, and test subsets containing 1400, 200, and 400 bridges, respectively. Splitting is performed by bridge identifier, keeping all records from each bridge within a single subset. The model with the lowest validation severity MSE, evaluated with all selected sensors available, is retained for testing. The networks are trained jointly using Adam with a learning rate of 10^−3^, a batch size of 64, and 100 epochs.

A separate model is trained for each prescribed failure rate. Measurement noise is evaluated during testing without retraining. Failure rates are treated as design scenarios rather than inputs to the gating network. During each forward pass, the gating network processes the bridge features to determine the number of sensors *k* and the objective weights. The selector network assigns an importance score to each of the N candidate locations, from which the *k* highest scoring locations are selected. The prediction network then estimates the damage severity from the selected structural responses, and the total loss is computed from the objectives defined in Section 2.7. During training, a random subset of the selected sensors is made unavailable according to the prescribed failure rate. The prediction network uses the surviving responses and availability indicators to estimate severity and compute the fault-tolerance loss.

### 2.9. Evaluation Procedure

We evaluated all the model’s experiments on an NVIDIA T4 GPU. Scenario-specific models are first evaluated on the same held-out test bridges with all selected sensors available. Cross-failure evaluation then tests each trained model at different failure rates without retraining. For each bridge, the selected layout remains fixed, and failures are applied only to its selected sensors.

Severity prediction performance was evaluated using Mean Absolute Error (MAE) and Root Mean Squared Error (RMSE):

(7) $$\mathrm{MAE}=\frac{1}{n}\sum_{i=1}^{n}\left|d_{i}-{\hat{d}}_{i}\right|$$

(8) $$\mathrm{RMSE}=\sqrt{\frac{1}{n}\sum_{i=1}^{n}{\left(d_{i}-{\hat{d}}_{i}\right)}^{2}}$$

where *d_i_* is the true minimum retained stiffness factor, ${\hat{d}}_{i}$ is its predicted value, and *n* is the number of test bridges.

MAE and RMSE are calculated on the original stiffness-factor scale. At each nonzero test failure rate, results are averaged over 30 random failure realizations.

Additionally, the reduction in the number of sensors relative to the full candidate set (*N* = 180) was calculated as:

(9) $$\mathrm{Sensor}\;\mathrm{reduction}\;(\%)=\left(1-\frac{\bar{k}}{N}\right)\times 100$$

where $\bar{k}$ is the mean number of selected sensors across the test bridges.

## 3. Results

In this section, we evaluate severity prediction, sensor count, and spatial coverage through scenario-specific training, cross-failure testing, baseline comparisons, out-of-distribution evaluation, candidate-count sensitivity, and measurement noise testing.

### 3.1. Severity Predictions After Scenario-Specific Training

This experiment evaluates scenario-specific optimization when the expected sensor failure rate is known during design. A separate model is trained for each prescribed rate. All models are evaluated on the same held-out test bridges with all selected sensors available. The prescribed rate represents an expected level of sensor unavailability considered during design. Training with simulated failures encourages the models to make sensor configurations that retain useful information when some of the selected sensors become unavailable. Table 2 shows the severity predictions after scenario-specific training.

We trained separate models with sensor failure rates ranging from 0% to 70%. The selected sensor count ranged from 60.36 to 101.03, corresponding to reductions of 43.87–66.47% relative to 180 candidate locations. Severity MAE ranged from 0.02389 to 0.03129, while RMSE ranged from 0.03487 to 0.03990. The model trained without failures achieved the lowest prediction errors. Models trained at 50% and 70% failures selected more sensors than those trained at 20% and 30%.

### 3.2. Cross-Failure Severity Prediction Without Retraining

We assessed the proposed method under changing failure conditions. Each scenario-specific model was tested at failure rates from 0% to 90% without retraining. For each model and bridge, the selected sensor layout is fixed; only the availability of sensors changes. All models were evaluated on the same 400 test bridges. Table 3 reports the mean severity MAE.

The cross-failure evaluation shows that the learned configurations can operate under failure conditions different from those used during training. The model trained without failures achieved the lowest MAE when all selected sensors were available. The model trained at a 30% failure rate achieved the lowest MAE at test failure rates of 10–50%. The model trained at 70% achieved the lowest mean MAE at 70% and 90% test failure rates.

### 3.3. Comparison Across Sensor Placement Methods

We compare the proposed method with uniform and random sensor placement using the same sensor number for each bridge. A full-sensor configuration using all 180 candidate locations is also included. Each method is evaluated on the same test bridges with all selected sensors available and with 50% test sensor failure. Each baseline layout uses a separately trained predictor with the same architecture as the proposed predictor. Baseline predictors are trained without simulated failures. Table 4 reports MAE and RMSE for each training scenario and its 50% test sensor failure.

The proposed method achieved lower MAE and RMSE than uniform and random placement in all reported comparisons, using the same sensor number. It used 43.87–66.47% fewer sensors than the full set of 180 candidate locations. These results illustrate a trade-off between sensor count and severity prediction performance. Performance under sensor failures reflects the combined contribution of the selected layouts and the proposed method’s failure-aware training, while baseline predictors were trained without simulated failures.

### 3.4. Comparison with Global Severity-Oriented Ranking

We compare the proposed method with a global severity-oriented ranking baseline that learns a shared ranking of candidate locations using severity-prediction error. Unlike the proposed bridge-specific selector, this baseline uses the same ranking across bridges. The number of selected sensors is matched to the proposed method for each bridge. Both methods use the same predictor architecture and are trained without simulated failures. They are evaluated on the same held-out bridges at 0% and 50% test sensor failure without retraining.

Table 5 shows the severity MAE and RMSE together with spatial coverage metrics. Coverage is evaluated for the installed layouts before sensor failures, using the sum of squared gaps and the largest uncovered interval, including the intervals at both ends of the bridge.

With all selected sensors available, the methods achieved similar prediction errors. Global ranking produced a slightly lower MAE, while the proposed method produced a slightly lower RMSE. At 50% sensor failure, the proposed method achieved slightly lower MAE and RMSE.

Additionally, the proposed method achieved a mean squared-gap sum of 0.01531, compared with 0.03420 for global ranking. Its mean largest uncovered interval was 3.09% of the bridge span, compared with 9.56%. These results show that the proposed method balances severity prediction with more even spatial coverage at matched sensor counts.

### 3.5. Generalization to Bridge Lengths Outside the Training Range

We evaluated the frozen model on 100 newly generated bridges within the training length range (80–150) and 100 bridges with lengths outside this range (155–180). No retraining was performed. Within each matched pair, only bridge length changed; material properties, cross-sectional geometry, damage factors, and load draws were identical. All selected sensors were available during evaluation.

Table 6 shows that MAE increased from 0.02403 to 0.02647, while RMSE increased from 0.03589 to 0.03718. The mean selected sensor count increased from 73.10 to 76.37. The OOD layouts also had lower squared-gap sums and smaller normalized uncovered intervals alongside the increased sensor count.

### 3.6. Sensitivity to the Number of Candidate Locations

To examine whether the results depend on candidate location discretization, we evaluated the framework using *N* = 90, 180, and 360 locations sampled uniformly along the bridge. A separate model was trained for each candidate count using the same bridge split, random seed, training settings, and minimum sensor count of five. All models were trained without simulated sensor failures and evaluated on the same 400 held-out bridges with all selected sensors available. Table 7 shows severity MAE, RMSE, mean selected sensor number, and sensor reduction relative to each candidate set.

Increasing the candidate count from 90 to 180 reduced both severity MAE and RMSE, while the mean selected sensor count increased from 35.52 to 74.4. Increasing the candidate count further to 360 produced similar MAE and slightly lower RMSE but increased the mean selected count to 163.21. These results support the use of 180 candidate locations as a compromise between prediction error and sensor count within the evaluated settings.

### 3.7. Severity Prediction Under Gaussian Measurement Noise

We evaluated the L2O-OSP model trained at 0% failure under additive Gaussian measurement noise, without retraining. The selected layouts remained fixed, and all selected sensors remained available. Noise was added only to the selected responses:

$$r_{b,i,c}^{\mathrm{noise}}=r_{b,i,c}+\in_{b,i,c};\;\in_{b,i,c}\sim ℵ\left(0,\alpha^{2}s_{c}^{2}\right).$$

where *r_b_*_,*i*,*c*_ is the response channel c at selected location i for bridge b, and *s_c_* is its standard deviation estimated from the training data, and α controls the noise level. The reported noise level is 100α%. Results at each nonzero noise level are average over 30 random realizations.

Table 8 showed that MAE and RMSE increased gradually with noise level. At 10% noise, MAE increased from 0.02389 to 0.02519, while RMSE increased from 0.03487 to 0.03697.

## 4. Discussion

The results demonstrate that the proposed L2O-OSP framework generates bridge dependent sensor configurations while simultaneously considering damage prediction accuracy, sensor deployment, spatial coverage, and prediction under sensor failures. The separately trained models selected between 60.36 and 101.03 sensors on average, corresponding to reductions of 43.87–66.47% relative to 180 candidate locations. Their prediction errors with all selected sensors available varied across training scenarios, showing that the training failure rate influenced the resulting trade-off.

Cross-failure evaluation separates the scenario-specific optimization from performance under changing sensor availability. Each trained model was tested without retraining or changes to its selected layout. MAE increased with test failure rate for every model. The model trained at a 30% failure rate achieved the lowest mean MAE at test failure rates of 10–50%, whereas the model trained at 70% achieved the lowest mean MAE at 70% and 90% test failure. No model performed best at every test failure rate. The models also used different numbers of sensors, which should be considered when comparing errors.

Additionally, the cross-failure results suggest a benefit from training with simulated sensor failures. At 70% and 90% test failure, every failure-trained model achieved lower MAE than the model trained without failure. The model trained at 70% failure selected the most sensors and achieved the lowest MAE under these severe conditions, which illustrates a trade-off between deployment cost and performance under sensor loss.

The comparison with global severity-oriented ranking shows the role of spatial coverage in the placement decision. At similar sensor counts, the methods achieved similar severity-prediction errors, while L2O-OSP produced more evenly distributed layouts and smaller uncovered intervals. This shows that coverage can be improved while maintaining comparable severity prediction performance. Additionally, the coverage results support the value of considering several objectives together. Moreover, L2O-OSP achieved lower prediction errors than uniform and random placement at matched sensor counts.

Furthermore, the candidate-count analysis shows that a denser set of possible locations does not always improve prediction. Increasing the candidate count from 90 to 180 reduced both MAE and RMSE. Increasing it further to 360 gave similar MAE and slightly lower RMSE but selected more than twice as many sensors on average. These results support 180 candidates as a practical compromise within the tested settings. Since the selected sensor count and network dimensions also changed, the results reflect their combined effect rather than candidate spacing alone.

Additionally, Gaussian noise was added to the measurements from fixed sensor layouts, without retraining. Prediction errors increased gradually, with MAE rising from 0.02389 to 0.02519 at the highest noise level. These results show how the trained predictor and selected layouts perform as measurement noise increases.

Finally, the length OOD experiments showed a modest increase in prediction error for bridges outside the training length range. The model selected slightly more sensors for these bridges, alongside smaller coverage gaps. These results provide evidence of generalization to longer bridges within the same simulated structural family.

However, the present study has several limitations. First, we evaluate the framework using numerically generated bridge configurations and simulated degradation scenarios. Uncertainty in material properties, support conditions, geometry, loading, and damage representation may change the structural responses and affect both sensor selection and severity prediction. Although the dataset includes parameter variations, these do not fully represent differences between simulated and real bridge behavior. The OOD experiment uses the same modeling assumptions and therefore does not replace experimental validation. Validation with measured bridge responses, supported where needed by finite element updating, is necessary for practical applicability.

Second, separate models are trained for prescribed failure rates, while measurement noise is evaluated on fixed layouts without retraining. Sensor loss and additive Gaussian noise represent only some monitoring disturbances. Future work could consider uncertain or correlated failures, sensor bias, drift, and gain errors, together with a single model conditioned on degradation levels.

Third, the present evaluation is limited to the structural configurations, loading conditions, and modeling assumptions presented in the generated dataset. Generalizing to other structural systems, boundary conditions, loading, and nonlinear structural behavior requires additional investigations. Further work should address these limitations through experimental and field validation and broader structural datasets.

To conclude, the proposed L2O-OSP framework for balancing sensor placement objectives presents several limitations and strengths compared to baseline methods. Table 9 summarizes the objectives, strengths, and limitations of the implementations evaluated in this study.

More broadly, this study supports the broader research direction of connecting OSP with the monitoring objective. OSP and damage assessment are conventionally treated as sequential and independent steps of the monitoring system’s design, with the sensor configuration determined before the performance of the following damage assessment model is considered. When placement and damage assessment are designed separately, the selected layout may not account directly for the prediction task. In L2O-OSP, severity prediction error is integrated into the multi-objective assessment of the selected sensor configurations, along with spatial coverage, sensor deployment cost, and fault tolerance.

This target is the minimum retained stiffness factor, representing the most severe element-level stiffness reduction. Different damage patterns can share this value, so the severity objective does not distinguish their location or extent. Future work could extend the prediction task to damage location or extent. XAI has also been used to examine parameter effects in models trained on FE-generated tunnel data [43]. Similarly, XAI could be used to investigate how bridge characteristics influence the sensor counts and objective weights produced by L2O-OSP.

## 5. Conclusions

In this paper, we proposed L2O-OSP, a learning-based optimal sensor placement framework for structural health monitoring. The framework integrates damage severity prediction, sensor deployment, spatial coverage, and fault tolerance within a multi-objective formulation. A gating mechanism determines sensor numbers and objective weights based on structural and loading characteristics. The model trained without sensor failures selected an average of 74.40 sensors from 180 candidate locations and achieved a severity MAE of 0.02389 with all selected sensors available.

Cross-failure testing evaluated the trained models without retraining or changes to their selected layouts. Compared with global severity-oriented ranking at matched sensor counts, L2O-OSP achieved similar severity-prediction errors and more even spatial coverage. Additional experiments assessed sensitivity to candidate count and Gaussian measurement noise, together with generalization to bridge lengths outside the training range.

Overall, these results demonstrate the potential of integrating sensor placement with the damage prediction objective without treating them as two separate tasks. Validation remains limited to simulated bridges. Future work will focus on experimental and field validation and evaluation across more structural characteristics and loading conditions.

## Figures and Tables

**Table 1:** Parameters and ranges used for the generation of the numerical bridge dataset.

Parameters	Range/Value
Number of bridge configurations	2000
Span length	80–150 m
Number of finite elements	800–1500
Elastic modulus E	30 ± 5 GPa
Cross-sectional area A	3.5–6.0 m^2^
Moment of inertia I	1.5–4 m^4^
Static load	100–200 kN
Traffic load	80–250 kN
Traffic load positions	15
Wind speed	15–38 m/s
Damage patterns	Localized (70%); distributed (30%)
Localized damage extent	5–15% of elements
Localized stiffness factor	0.6–1.0
Distributed stiffness factor	0.7–1.0
Localized case perturbation	±0.02%

**Table 2:** Severity prediction after scenario-specific training.

Training Failure Rate	Mean Sensors Selected	MAE	RMSE	Sensor Reduction
0%	74.40	0.02389	0.03487	58.67%
10%	70.92	0.02528	0.03598	60.60%
20%	60.36	0.02557	0.03532	66.47%
30%	61.38	0.02486	0.03635	65.90%
50%	80.22	0.03129	0.03990	55.43%
70%	101.03	0.02868	0.03791	43.87%

**Table 3:** Mean severity MAE across training and test sensor failure rates without retraining.

Training Failure Rate	Mean Sensors Selected	Test: 0%	Test: 10%	Test: 20%	Test: 30%	Test: 50%	Test: 70%	Test: 90%
0%	74.40	0.02389	0.02661	0.02926	0.03146	0.03557	0.03836	0.04053
10%	70.92	0.02528	0.02656	0.02821	0.03013	0.03397	0.03729	0.03993
20%	60.36	0.02557	0.02701	0.02862	0.03025	0.03348	0.03608	0.03821
30%	61.38	0.02486	0.02611	0.02775	0.02946	0.03306	0.03641	0.03933
50%	80.22	0.03129	0.03189	0.03249	0.03333	0.03534	0.03720	0.03893
70%	101.03	0.02868	0.02958	0.03053	0.03136	0.03351	0.03511	0.03519

**Table 4:** Severity prediction comparison with uniform, random, and full sensor configurations at 0% and 50% test failures.

Training Failure Rate	Method	Mean Sensors Selected	MAE: 0% Failure	RMSE: 0% Failure	MAE: 50% Failure	RMSE: 50% Failure
10%	Proposed	70.92	0.02528	0.03598	0.03397	0.04084
0%	Uniform sensor placement	70.92	0.04027	0.04453	0.04033	0.04421
0%	Random sensor placement	70.92	0.03869	0.04523	0.04161	0.04605
0%	Full sensors	180	0.02768	0.03602	0.03590	0.04318
20%	Proposed	60.36	0.02557	0.03532	0.03348	0.04054
0%	Uniform sensor placement	60.36	0.04047	0.04425	0.04019	0.04406
0%	Random sensor placement	60.36	0.04032	0.04413	0.04005	0.04406
0%	Full sensors	180	0.02768	0.03602	0.03590	0.04318
30%	Proposed	61.38	0.02486	0.03635	0.03306	0.04005
0%	Uniform sensor placement	61.38	0.03925	0.04496	0.04020	0.04428
0%	Random sensor placement	61.38	0.03931	0.04425	0.03987	0.04421
0%	Full sensors	180	0.02768	0.03602	0.03590	0.04318
Proposed		80.22	0.03129	0.03990	0.03534	0.04133
0%	Uniform sensor placement	80.22	0.03691	0.04630	0.03795	0.04462
0%	Random sensor placement	80.22	0.04104	0.04438	0.04056	0.04420
0%	Full sensors	180	0.02768	0.03602	0.03590	0.04318
70%	Proposed	101.03	0.02868	0.03791	0.03351	0.04076
0%	Uniform sensor placement	101.03	0.03700	0.04302	0.03740	0.04427
0%	Random sensor placement	101.03	0.04164	0.04448	0.04063	0.04421
0%	Full sensors	180	0.02768	0.03602	0.03590	0.04318

Note: Uniform and random layouts match the proposed method’s sensor count for each bridge.

**Table 5:** Severity prediction and spatial coverage with global severity-oriented ranking at the same sensor numbers.

Method	Mean Sensors Used	MAE: 0% Failure	RMSE: 0% Failure	MAE: 50% Failure	RMSE: 50% Failure	Mean Squared-Gap Sum	Mean Largest Gap (%)
Proposed	74.40	0.02389	0.03487	0.03557	0.04357	0.01531	3.09
Global severity-oriented ranking	74.40	0.02371	0.03489	0.03608	0.04446	0.03420	9.56

**Table 6:** Severity prediction and spatial coverage within and outside the training bridge length range.

Domain	Bridges	MAE	RMSE	Mean Selected Sensors	Mean Squared-Gap Sum	Mean Largest Gap (%)
New In-distribution length	100	0.02403	0.03589	73.10	0.01569	3.17
Out-of-distribution Length	100	0.02647	0.03718	76.37	0.01483	2.90

**Table 7:** Sensitivity of severity prediction and sensor selection to the number of candidate locations.

Candidate Locations N	Mean Selected Sensors	MAE	RMSE	Sensor Reduction (%)
90	35.52	0.03325	0.04132	60.53
180	74.40	0.02389	0.03487	58.67
360	163.21	0.02439	0.03422	54.66

**Table 8:** Severity prediction under Gaussian noise with fixed sensor layouts and no retraining.

Noise Level (%)	Mean Sensors Selected	MAE	RMSE
0.0	74.40	0.02389	0.03487
1.0	74.40	0.02391	0.03490
5.0	74.40	0.02425	0.03545
10.0	74.40	0.02519	0.03697

**Table 9:** Summary of the objectives, strengths, and limitations of the implementations evaluated in this study.

Method	Placement Objective	Strength	Limitation
L2O-OSP	Severity error, coverage, sensor count, and error under failures	Bridge-specific layouts and sensor counts	Requires training and FE responses at all candidates; separate models for prescribed failure rates
Global severity-oriented ranking	Severity prediction error	Task-oriented shared ranking	Shared ranking; larger coverage gaps in the evaluated layouts
Uniform placement	Even spatial spacing	Evenly distributed sensors	Does not guarantee response informativeness
Random placement	Random selection at a prescribed count	Easy implementation	Does not guarantee even coverage

## Data Availability

The data supporting the findings of this study are available from the corresponding author upon reasonable request.

## References

[1] Svendsen B.T., Frøseth G.T., Øiseth O., Rønnquist A. (2022). A data-based structural health monitoring approach for damage detection in steel bridges using experimental data. J. Civ. Struct. Health Monit..

[2] Sun Z., Mahmoodian M., Sidiq A., Jayasinghe S., Shahrivar F., Setunge S. (2025). Optimal Sensor Placement for Structural Health Monitoring: A Comprehensive Review. J. Sens. Actuator Netw..

[3] Hassani S., Dackermann U. (2023). A Systematic Review of Optimization Algorithms for Structural Health Monitoring and Optimal Sensor Placement. Sensors.

[4] Kyung J., An J.-H., Eun H.-C. (2024). Sensor Layout Design for Structural Health Monitoring. Civ. Eng. J..

[5] Lee E.-T., Eun H.-C. (2022). Optimal Sensor Placement in Reduced-Order Models Using Modal Constraint Conditions. Sensors.

[6] Das S., Saha P., Das B.B., Gomez C.P., Mohapatra, Benu G. (2022). Performance of Optimal Sensor Placement Strategies for Damage Detection in Civil Engineering. Recent Developments in Sustainable Infrastructure (ICRDSI-2020)—Structure and Construction Management.

[7] Worden K., Burrows A.P. (2001). Optimal sensor placement for fault detection. Eng. Struct..

[8] Chioccarelli E., Giunta D., De Angelis A., Bilotta A. (2024). Discussing optimal sensor placement for ambient vibration test of an existing bridge. Eng. Struct..

[9] Tan Y., Zhang L. (2020). Computational methodologies for optimal sensor placement in structural health monitoring: A review. Struct. Health Monit..

[10] Ostachowicz W., Soman R., Malinowski P. (2019). Optimization of sensor placement for structural health monitoring: A review. Struct. Health Monit..

[11] Mallardo V., Aliabadi M.H. (2013). Optimal sensor placement for structural, damage and impact identification: A review. Struct. Durab. Health Monit..

[12] Wang Y., Chen Y., Yao Y., Ou J. (2023). Advancements in Optimal Sensor Placement for Enhanced Structural Health Monitoring: Current Insights and Future Prospects. Buildings.

[13] Kammer D.C. (1991). Sensor placement for on-orbit modal identification and correlation of large space structures. J. Guid. Control Dyn..

[14] Yi T.-H., Li H.-N., Gu M. (2011). A new method for optimal selection of sensor location on a high-rise building using simplified finite element model. Struct. Eng. Mech. An. Int. J..

[15] Sun Z., Manuel M.I., Mahmoodian M., Sidiq A., Chan R.W.K., Jayasinghe S.C., Shahrivar F., Setunge S. (2024). Optimal sensor placement applications for bridge structural health monitoring using the effective independence method. Bridge Maintenance, Safety, Management, Digitalization and Sustainability.

[16] Chai W., Yang Y., Yu H., Yang F., Yang Z. (2022). Optimal sensor placement of bridge structure based on sensitivity-effective independence method. IET Circuits Devices Syst..

[17] Meo M., Zumpano G. (2005). On the optimal sensor placement techniques for a bridge structure. Eng. Struct..

[18] Li D.S., Li H.N., Fritzen C.P. (2007). The connection between effective independence and modal kinetic energy methods for sensor placement. J. Sound Vib..

[19] Masciotta M.G., Barontini A., Chaves E., Mendes N., Brando G., Lourenço P.B. (2025). Modal-based multi-criteria optimization of sensor placement techniques for dynamic monitoring of bridges. Int. J. Bridge Eng. Manag. Res..

[20] Mghazli M.O., Zoubir Z., Nait-Taour A., Cherif S., Lamdouar N., El Mankibi M. (2023). Optimal sensor placement methodology of triaxial accelerometers using combined metaheuristic algorithms for structural health monitoring applications. Structures.

[21] Van T.N., Quang M.T. (2026). Damage Detection in Bridges by Hybrid Particle Swarm Optimization–Simulated Annealing (PSO–SA) Approach. IEEE Access.

[22] Civera M., Pecorelli M.L., Ceravolo R., Surace C., Zanotti Fragonara L. (2021). A multi-objective genetic algorithm strategy for robust optimal sensor placement. Comput.-Aided Civ. Infrastruct. Eng..

[23] Saleem M.M., Jo H. (2021). Multi-objective sensor placement optimization for structural response estimation under spatially varying dynamic loading of bridges. Adv. Struct. Eng..

[24] Cha Y.-J., Agrawal A.K., Kim Y., Raich A.M. (2012). Multi-objective genetic algorithms for cost-effective distributions of actuators and sensors in large structures. Expert Syst. Appl..

[25] He C., Xing J., Li J., Yang Q., Wang R., Zhang X. (2013). A Combined Optimal Sensor Placement Strategy for the Structural Health Monitoring of Bridge Structures. Int. J. Distrib. Sens. Netw..

[26] Zhang C., Chun Q., Lin Y. (2025). Optimal sensor placement and structural health monitoring methods of ancient stone bridges based on an improved genetic algorithm: Taking Lugou Bridge as an example. Measurement.

[27] Chen T., Chen X., Chen W., Heaton H., Liu J., Wang Z., Yin W. (2022). Learning to Optimize: A Primer and A Benchmark. J. Mach. Learn. Res..

[28] Castera C., Ochs P. (2025). From Learning to Optimize to Learning Optimization Algorithms. Proceedings of the 28th International Conference on Artificial Intelligence and Statistics.

[29] Meng X., Zhang H., Jia K., Li H., Huang Y. (2023). Optimal sensor placement for structural health monitoring based on deep reinforcement learning. Smart Struct. Syst..

[30] Sajedi S., Liang X. (2022). Deep generative Bayesian optimization for sensor placement in structural health monitoring. Comput.-Aided Civ. Infrastruct. Eng..

[31] Shi S., Du D., Mercan O., Kalkan E., Wang S. (2024). A novel data-driven sensor placement optimization method for unsupervised damage detection using noise-assisted neural networks with attention mechanism. Mech. Syst. Signal Process..

[32] Zhong Q., Wei S., Huang Y., Jia K., Li H. (2025). Optimal placement of multiple types of sensors based on Bayesian system identification using heuristic-enhanced deep reinforcement learning. Eng. Struct..

[33] Tang K., Yao X. (2024). Learn to optimize—A brief overview. Natl. Sci. Rev..

[34] Wang Z., Li H.-X., Chen C. (2020). Reinforcement Learning-Based Optimal Sensor Placement for Spatiotemporal Modeling. IEEE Trans. Cybern..

[35] Zhou G., Long W., Shen A., Zhang J., Yang J. (2025). Robustness-oriented optimal sensor placement for structural monitoring considering sensor failures. J. Southeast Univ. (Engl. Ed.).

[36] Xu X., Liu X., Jin Y., Zhang Q., Lan J., Hou Z., Ma T., Forde M.C. (2026). A deep learning-empowered data anomaly detection method based on a symmetric dot pattern for bridge monitoring data. Struct. Health Monit..

[37] Rillo V., De Angelis A., Maddaloni G., Rainieri C., Gentile C., Aenlle López M. (2024). A Framework to Define an Effective Structural Health Monitoring (SHM) System Using the Data from OMA Test. Proceedings of the 10th International Operational Modal Analysis Conference (IOMAC 2024).

[38] Chang M., Pakzad S.N. (2014). Optimal Sensor Placement for Modal Identification of Bridge Systems Considering Number of Sensing Nodes. J. Bridge Eng..

[39] Zhou Z., Chen M., Zhou W., Xiao Q. (2026). PCIM-DTA: Pairwise conditional interaction modeling for drug–target affinity prediction under cold-start scenarios. Bioinformatics.

[40] Zhou Z., Chen M., Zhou W., Xiao Q. (2026). Similarity-Adaptive interaction routing for Drug–Target affinity prediction. J. Biomed. Inform..

[41] Mazzoni S., McKenna F., Scott M.H., Fenves G.L. (2006). OpenSees command language manual. Pac. Earthq. Eng. Res. Cent..

[42] Japan Road Association (2019). Highway Bridge Specifications (Part I: Common).

[43] Wu G., Tan Y., Xu Z., Yuan Z., Zhang J., He Y., Wang C. (2026). XAI-Aided Method for Accurately Assessing the Stability of Unlined Double Tunnels in Rock Masses. Transp. Geotech..

